# The Free Radical Diseases of Prematurity: From Cellular Mechanisms to Bedside

**DOI:** 10.1155/2018/7483062

**Published:** 2018-07-24

**Authors:** Serafina Perrone, Antonino Santacroce, Mariangela Longini, Fabrizio Proietti, Francesco Bazzini, Giuseppe Buonocore

**Affiliations:** Department of Molecular and Developmental Medicine, University of Siena, Siena, Italy

## Abstract

During the perinatal period, free radicals (FRs) are involved in several physiological roles such as the cellular responses to noxia, the defense against infectious agents, the regulation of cellular signaling function, and the induction of a mitogenic response. However, the overproduction of FRs and the insufficiency of an antioxidant mechanism result in oxidative stress (OS) which represents a deleterious process and an important mediator of damage to the placenta and the developing fetus. After birth, OS can be magnified by other predisposing conditions such as hypoxia, hyperoxia, ischemia, hypoxia ischemia-reperfusion, inflammation, and high levels of nonprotein-bound iron. Newborns are particularly susceptible to OS and oxidative damage due to the increased generation of FRs and the lack of adequate antioxidant protection. This impairment of the oxidative balance has been thought to be the common factor of the so-called “free radical related diseases of prematurity,” including retinopathy of prematurity, bronchopulmonary dysplasia, intraventricular hemorrhage, periventricular leukomalacia, necrotizing enterocolitis, kidney damage, and oxidative hemolysis. In this review, we provide an update focused on the factors influencing these diseases refining the knowledge about the role of OS in their pathogenesis and the current evidences of such relationship. Mechanisms governing FR formation and subsequent OS may represent targets for counteracting tissue damage.

## 1. Introduction

Each molecule is characterized by a particular concentration of electrons that establish its own redox state. When specific conditions occur, the redox state can be altered to lower or higher levels thus forming free radicals (FRs) [1]. FRs are highly reactive substances that are capable to start self-amplified chain reactions causing cellular dysfunction and damage. Many antioxidant enzymes exist to counteract this propagation, and when the production of FRs exceeds the capacity of scavenger defenses, an oxidative stress (OS) occurs [2].

In the perinatal period, a properly controlled oxidative species production has been proven to be a necessary factor [3]. After fertilization, the beneficial effects of FRs occur at low/moderate concentrations and involve physiological roles in sperm capacitation, acrosome reaction, sperm-egg interaction, and gamete fusion [4, 5]. Until the beginning of the second trimester, fetal development takes place in a low-oxygen environment presumably to protect the embryo, which is highly sensitive to reactive oxygen species (ROS) [6]. Subsequently, due to the placental maturation, a threefold rise in the oxygen concentration causes an exponential increase of ROS [7]. In this phase, ROS regulate gene transcription and downstream activities such as trophoblast proliferation, invasion, and angiogenesis. OS-induced apoptosis influences placental vascular modifications [8], induces autophagy, and together ensures the normal cellular turnover until the late gestation [7]. The placenta adapts to the increase in ROS generation by modulating hypoxia-inducible factor 1*α* (HIF-1*α*) and increasing cellular antioxidant levels [9]. Under normal conditions, such adaptation mechanisms must occur to ensure a proper fetal development [10]. However, in a scenario where OS is abnormally increased, chelation mechanisms may be insufficient and damage affecting both the fetus and the mother can be observed. Many studies have shown a relationship between increased OS levels and several pregnancy diseases, including defective embryogenesis, spontaneous abortion, preeclampsia, intrauterine growth restriction, gestational diabetes mellitus, premature rupture of membranes, and minor congenital abnormalities [3, 9, 10–12].

The intrauterine environment and redox state can also play a role for future diseases in the child such as obesity and diabetes mellitus and hypertension in adulthood [13–15]. Finally yet importantly, an increased oxidative level in the perinatal life may trigger a deleterious state of OS in the newborn, especially in preterm infants, which is capable to activate underlying mechanisms that lead to the onset of the so-called “oxidative stress-related diseases in newborn” [16–18]. The present review intends to make a journey into the free radical chemical biology and to update the current knowledge about the role of OS in the pathogenesis of such gestational, fetal, and neonatal diseases.

### 1.1. Free Radical Chemical Biology

Free radical reactions are a normal occurrence in living organisms, and ROS are deeply involved in signaling molecules to regulate a wide variety of physiological events.

**Figure 1:**
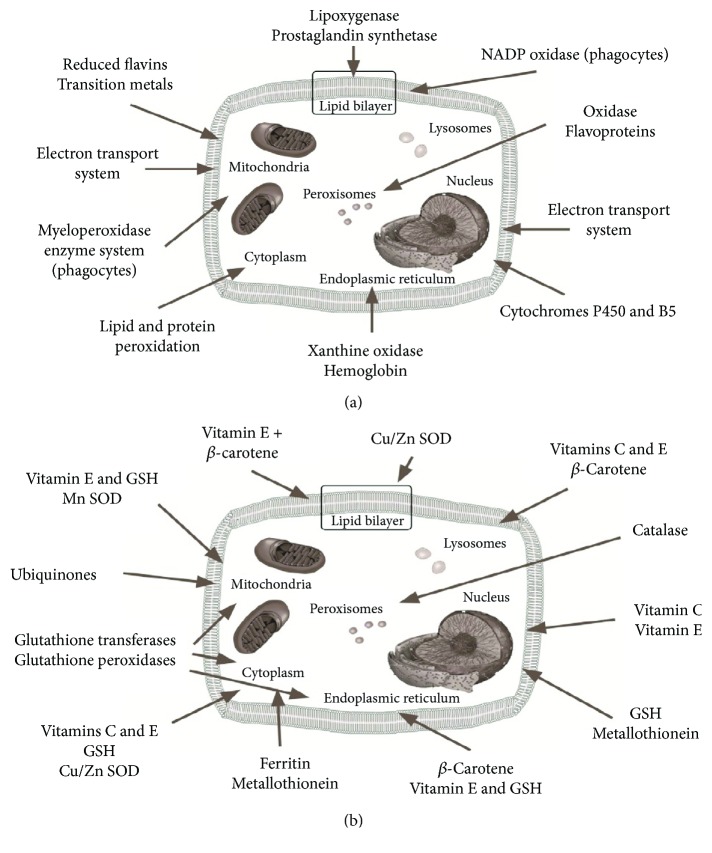
Cellular sources of free radicals (a); antioxidant protection within the cell (b).

There are different FR species: oxygen-centred radicals (ROS), nitrogen-centred radicals (RNS), carbon-centred radicals, and sulphur-centred radicals [19]. Intracellular generation of ROS can occur as a byproduct with mitochondria, peroxisomes, cytochrome P450, and other cellular elements (Figure 1(a)). ROS generation by mitochondria is a highly variable and depends both from metabolic conditions and the intramitochondrial balance between oxidative and antioxidative factors.

Under physiologic conditions, approximately 98% of O_2_ undergoes a complete reduction to form H_2_O_2_. Approximately, 2% of electrons will leak causing a partial reduction of O_2_-producing ROS [20]. The monovalent reduction of O_2_ produces superoxide anion (O^−^), and monovalent reduction of O^−^ generates hydrogen peroxide (H_2_O_2_). A third monovalent reduction generates the highly reactive hydroxyl radical (OH^•^) [21, 22].

Any damage to the energy-producing machinery of the mitochondria will result in superoxide accumulation, and any process that results in depletion of antioxidant defense will result in the default conversion of superoxide to even more oxygen reactive species.

Other important FR cellular sources are the following: the activity of monoamine oxidase, which deaminates biogenic amines and produces hydrogen peroxide (H_2_O_2_); the purine catabolism by xanthine oxidase (XO), producing superoxide anion radical (O_2_^−^); the Haber-Weiss and Fenton reactions by nonprotein-bound iron (NPBI) producing the hydroxyl radical (OH); peroxynitrite (ONOO^–^) generator enzymes, such as xanthine oxidoreductase, nicotinamide adenine dinucleotide phosphate oxidase (Nox), nitric oxide synthase (NOS), and heme oxygenase (HO) [23]; and the inflammatory response where the production of H_2_O_2_ and O_2_^•−^ increases in white blood cells and Kupffer cells due to NADPH-dependent oxidase system, coupled to the action of superoxide dismutase (SOD).

Aerobic organisms have developed well-integrated antioxidant defenses to enable them to handle and scavenge FRs [24] (Figure 1(b)). These defenses include antioxidant enzymes and low molecular weight antioxidant compounds like vitamins A, E, and C, beta-carotene, lipoic acid, and glutathione. The antioxidant enzymes, like SOD, catalase, and GPX, have the capacity to scavenge the levels of ROS produced ever in physiological conditions [25]. Under ischemic conditions, these antioxidant enzymes fail to protect tissue from oxidative damage because of the overproduction of oxygen radicals and consumptions of antioxidant defense [25].

Once generated, the condition of OS may perpetuate the damage to all components of the cell, including proteins, lipids, and DNA. F2-isoprostanes (F2-IsoPs) are prostaglandin F2-like compounds derived by the FR peroxidation of arachidonic acid. They are recognized as reliable marker of lipid peroxidation. Docosahexaenoic acid (DHA), a major component in neuronal membranes, oxidizes both in vitro and in vivo to form F2-IsoP-like compounds termed F4-neuroprostanes (F4-NPs) [16, 26–29]. An oxygen insertion step diverts intermediates from the IsoP pathway to form isofurans (IsoFs) that contain a substituted tetrahydrofuran ring. Because of this differential method of formation, it has been focused that oxygen concentration can affect lipid peroxidation profile. Like the IsoPs, the IsoFs are chemically and metabolically stable so are well suited to act as *in vivo* biomarkers of oxidative damage. The NPs are the only quantitative *in vivo* biomarker of oxidative damage that is selective for neurons. An alternative pathway of oxidation of DHA brings to the formation of an IsoF-like compound termed neurofurans (NFs). Quantitative assessment of NFs in vivo reveals modulated formation under conditions of elevated and diminished OS [28]. Proteins are also vulnerable to FR attacks. Similar to lipid peroxides, altered protein molecules, such as protein peroxides and protein-bound reducing moieties, can act as traps for the chemical energy released by FR. Then they can initiate further radical chain reactions, thus enhancing the damage. Advanced oxidative protein products (AOPP) are reliable markers of the degree of protein damage in OS [30, 31].

In term of increased NPBI concentration, the link between increased FR release and OS during fetal/neonatal asphyxia has emerged with reports of increased plasma F2-isoprostanes and advanced oxidative protein products (AOPP), as indices of lipid and protein oxidation, in cord blood [27, 32].

## 2. Oxidative Stress in the Prenatal Period

Embryos and fetuses have a relative immaturity of the antioxidant system that facilitates the exposure to the damaging effects of an OS condition [12]. ROS highly affect embryo and fetus development, thus causing different diseases with a common pathophysiology based on antioxidant impairment and FR overproduction.

Some conditions of pregnancy are specific triggers for an overload of FRs, thus setting an adverse intrauterine environment with subsequent fetal development impairment [33, 34]. Morphological and immunohistochemical analyses show an increased oxidative damage in placental tissues obtained from early spontaneous abortion compared with normal controls [35]. On the other hand, impaired extravillous trophoblast invasion and insufficient uterine artery remodeling, associated with the onset of preeclampsia (PE), lead to highly resistant spiral arteries and to ischemia/reperfusion of placenta [36, 37]. Oxidase activity was detected and confined to the microvillus membrane of syncytiotrophoblast and might be abnormally regulated in PE pregnancies [33]. A deregulation of phospholipases A2 could potentially be implicated in freeing F2-isoP, which could participate in local hypertension observed in the PE placenta through the thromboxane pathway. Indeed, free F2-isoP was found to be significantly higher in preeclamptic woman than normotensive controls [38]. PE is not only an endothelial disease but also a consequence of a wider range of systemic inflammatory network responses [39]. Activated macrophages, neutrophils, and Th1 cells can infiltrate into renal and other tissues in women with PE [39]. Systemic inflammation and circulating antiangiogenic factors may ultimately end in multiorgan dysfunction if not controlled in a timely manner [40].

Early-onset PE is almost invariably associated with IUGR [34]. Despite having distinctive clinical manifestations, there is an accumulating evidence that the two pathologies have a common cause: an abnormal placental implantation [41]. This deficiency is thought to be associated with placenta underperfusion, which is a high risk factor for subsequent OS [42]. In IUGR, OS may hinder the placental neutral amino acid transport and may reduce the glucose accumulation in the syncytiotrophoblast, all of which decrease the uptake of critical materials for fetal development [43]. The relationship between IUGR and OS was reported by several authors [11, 44, 45]. In particular, neonates with IUGR showed a significant deficiency in antioxidant defenses as well as an increased lipid peroxidation [46]. F2-IsoP, which represents the main marker of the arachidonic acid peroxidation, was higher in pregnancies with fetal growth restriction and showed a moderate power on distinguish between normal and restricted growth fetuses, when tested on amniotic fluid [11].

FRs may also disrupt the amino acid binding to proteins and polyunsaturated fatty acids of lipid membranes, thus causing cell dysfunction, modification of chorioamniotic biology, and, finally, the premature rupture of membranes [11]. For confirmation, higher levels of F2-IsoP were found in amniotic fluid of mothers with premature rupture of membranes, compared with control pregnancies [47].

Diabetes and obesity may induce OS in pregnancy, which in turn may cause biochemical disturbances of the fetus and newborn [48, 49]. In diabetes mellitus, ROS are produced in excess due to the prolonged periods of hyperglycemia, which is known to cause nonenzymatic glycation of plasma proteins [23]. Moreover, glucose undergoes autooxidation, thus forming FR hydroxilic anions that overwhelm the antioxidant cellular responses. The high blood glucose levels can affect the surrounding vasculature, causing the endothelium to be more sensitive to FRs [50]. To date, in diabetes experimental models, an increased ROS generation was highlighted in the embryos, fetuses, and placentas [51–53]. This increase in intrauterine OS during embryonic and feto-placental development has been associated with an impaired organogenesis [54]. A detrimental state of OS may be inherited at birth by the intrauterine environment.

## 3. Free Radical-Related Diseases of the Newborn: Overview

Newborns are particularly prone to OS due to the exposure to conditions that can cause a burden of FRs. Preterms have weak antioxidant defenses that are not able to counteract the harmful effects of FRs. Moreover, frequent condition such as ischemia, hypoxia-reperfusion, infection, and inflammation are triggers capable to produce high levels of FRs, thus perturbing the normal redox balance and shifting cells into a state of OS [55]. Blood transfusions, increased levels of nonprotein-bound iron (NPBI), xenobiotics, drugs, and hyperoxia are other potential sources of FR generation.

Cellular, tissue, and organ damages, involving kidney, retina, lung, and bowel injuries, have been related with OS biomarker levels in cord blood [27], thus leading to the hypothesis of free radical-related diseases of prematurity. Intraventricular hemorrhage, retinopathy of prematurity, bronchopulmonary dysplasia, and necrotizing enterocolitis have been already included in this group of conditions [26]. Later in this work, the last evidences supporting the relationship between OS and diseases of prematurity will be updated. Moreover, other conditions such as kidney damage and hemolysis will be reviewed and discussed.

### 3.1. Intraventricular Hemorrhage

**Figure 2:**
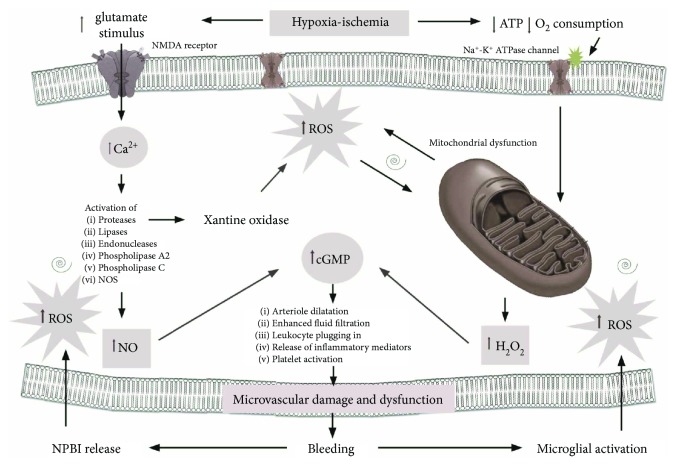
Schematic representation of vascular cerebral injury.

Intraventricular hemorrhage (IVH) in very preterm infants is a common disease associated with long-term consequences [56]. The hemorrhage typically involves the periventricular germinal matrix (GM). Pathogenesis of GMH-IVH is multifactorial, complex, and heterogeneous. An inherent fragility of the GM vasculature predisposes to hemorrhage, and fluctuation in the cerebral blood flow induces the rupture of blood vessels (Figure 2). Platelet or coagulation disorders might accentuate and perpetuate the hemorrhage [57]. The inherent fragility of the germinal matrix vasculature may be further exacerbated following hypoxia, but the precise effects on cerebral blood vessels remain poorly understood [58]. Recently, more detailed analyses have demonstrated the role of OS in this context [59]. During hypoxia, FR production increases, enhancing all the pathways implicated in microvascular damage and dysfunction. H_2_O_2_ and nitric oxide radicals (NO^•^) are able to activate the soluble enzyme guanylate cyclase, which catalyzes the formation of the cyclic “second messenger” guanosine monophosphate (cGMP). cGMP modulates the function of protein kinases, ion channels, and other important targets, leading to altered dilatation of arterioles, enhanced fluid filtration, leukocyte plugging in capillaries, and release of inflammatory mediators and platelet activation [60]. The oxidative events that trigger the initiation of bleeding into the germinal matrix promote a cascade leading to the disruption of tight junctions, to the increased blood-brain barrier permeability, and to microglial activation within the developing periventricular white matter. These events are mediated by cytokines (IL-1*β* and TNF-*α*), VEGF, and NO. Finally, reactive microglia release ROS, which in turn not only contribute to endothelial damage but also alter hemostasis and increase anaerobic metabolism [61]. In our previous study, we found increased plasma levels of total hydroperoxides (TH), advanced oxidation protein products (AOPP), and particularly NPBI in newborn who developed IVH [27]. Hypoxia and ischemia are the most important source of nonprotein-bound iron (NPBI) [62]. Moreover, the latter's conditions also supply redox-cycling iron, enhancing NPBI release into plasma [63]. Even higher increment of NPBI was described during reperfusion phase by erythrocyte releasing [64]. Due to low transferrin levels, the decreased transferrin iron-binding capacity, and low levels of ceruloplasmin and ferroxidase activities, premature infants may be particularly prone to an exaggerated generation of NPBI [65–68]. In this case, iron is capable of causing degeneration of endothelial cells [69]. Endothelial cell injury and dysfunction may additionally contribute to the inflammatory response and alteration in coagulation, through loss of normal endothelial NO production [70]. Other potential implications of iron overload are acute impairment of endothelium-dependent flow-mediated vasodilation [71], loss of tight junction proteins, degeneration of endothelial cells, and opening of the blood-brain barrier [69]. Separation of endothelial tight junctions, loss of endothelial attachment to the basement membrane, endothelial blebbing, and endothelial necrosis have been described in the cerebral vasculature following ischemic injury [72]. The progression of endothelial dysregulation can contribute to the ongoing pathogenesis of IVH.

### 3.2. Retinopathy of Prematurity

Retinopathy of prematurity (ROP) is the major cause of visual impairment and blindness in premature neonates worldwide [73]. ROP-associated blindness incidence has been reported to be lower than 10% of extremely preterm born children, but in some low- and middle-income countries, the incidence can reach the 40% [74, 75]. Normally, the peripheral retinal vascularization keeps developing until the fetus is near to full-term. ROP occurs in two phases: the vascular attenuation phase (phase I) and the fibrovascular proliferative phase (phase II). In phase I, a cessation of normal retinal vascularization is driven by hyperoxia, while in phase II, hypoxia renews vascularization. In both of these cases, VEGF plays a major role but in an opposite manner [76]. During hyperoxic phase I, VEGF is suppressed arresting the normal retinal vascularization and leading to the loss of some developing vessels. Later in retinal development, the oxygen need increases and a hypoxic condition arises. VEGF production begins in response to this hypoxia, thus giving rise to retinal neovascularization in the outer border, between vascularized and nonvascularized retinas [77]. The action of VEGF depends on insulin-like growth factor-1 (IGF-1) [78]. Fetal IGF-1 precipitously falls after premature birth and increases due to the newborn's maturation, thus contributing to the later neovascularization process.

It is not clear why some babies develop severe ROP whereas other babies with similar clinical characteristics do not progress to a severe stage. Genetic factors in addition to prematurity or environmental conditions may be responsible of the development and progression of ROP.

OS may represent a key mechanism in this different individual response, depending from each own redox state. Different from VEGF, OS acts in a continuum manner into ROP pathophysiology through intracellular ROS generation, which acts as signaling effectors. Several experimental studies have deepened our understanding of the role of oxidative and nitrosative compounds on ROP. In these studies, crosstalk among inflammatory, metabolic, and angiogenic pathways showed a trigger effect on pathologic or physiologic intracellular oxidative signaling mechanisms [79].

During phase I, oxygen and overall hyperoxia are fundamental factors, with a direct relationship between a high-oxygen saturation and ROP [77]. Nitrosative stress through ONOO^–^ generation may lead to hyperoxia-induced apoptosis [78] which was found to be implicated in neuroglial injury [79] and vaso-obliteration in “oxygen-induced retinopathy” (OIR) models [80]. Transcription factor, Nrf2, showed an antioxidant protection against hyperoxia-induced endothelial loss in OIR and increases avascular retina [81].

In phase II OIR models, hypoxia- or oxidative-induced factors mediated the overexpression of VEGF signaling through VEGF receptor 2 (VEGF2). Overactive VEGFR2 then triggered downstream signaling events that disoriented endothelial cell divisions and enabled them to grow outside the plane of the retina rather than within the retina [82–84]. In this context, ROS were mainly produced by Nox [85]. Various isoforms of Nox have been implicated, including Nox-1 [86], Nox-2 [87], and Nox-4 [88]. Controversially, Nox is the key enzyme in leukocytes which serves to fight invading microorganisms; hence, inhibiting Nox may have unwanted consequences. Nitric oxide (NO) can also have beneficial effects on endothelial cells as a vasodilator, but NO may be capable to generate ONOO^–^ in the presence of high concentrations of superoxide radical. Thus, we are presented with a double-edged sword and a balance between, or a specific targeting of, oxidative/nitrosative effects.

Clinical studies supported the risk of oxygen and hyperoxia in ROP pathogenesis, thus describing how O_2_ administration in delivery room was significantly associated with the development of ROP [89]. Very high concentrations of hypoxanthine, which is produced during hypoxia/reperfusion, were found in the eyes of babies at risk of developing ROP [90].

It has been shown that the retinal antioxidant and chelator enzyme content is low in ROP cases [91]. Enhancing endogenous or exogenous antioxidant power may help in counteracting OS-related injury [92, 93]. Protective effects have been shown by giving the potent antioxidant D-penicillamine and vitamin E [94], but several further studies are needed to properly analyze other promising antioxidant agents (Table 1).

### 3.3. Bronchopulmonary Dysplasia

Bronchopulmonary dysplasia (BPD) is the most common adverse respiratory outcome in very preterm neonates [95, 96]. Originally, “old” BPD was defined based on lung injury occurring in premature infants born between 29 and 32 weeks of postmenstrual age (PMA), due to their respiratory distress syndrome (RDS) requiring oxygen supplementation and especially mechanical ventilation [97]. Later, the introduction of maternal corticosteroids [98] and surfactant replacement therapy [99] resulted in a different disease phenotype (“new” BPD) that was seen in younger preterm infants (below 29 weeks PMA), based on impaired alveolar and capillary development of the immature lungs [96]. Depending on the cohort and definition used, the overall incidence variates between 5 and 68% and increases significantly with declining gestational age [100]. Prematurity, oxygen toxicity, inflammation, mechanical ventilation, and surfactant deficit are major factors contributing to the pathogenesis [101]. Recently, much more importance has given to prenatal environment [102]. In example, preeclampsia alone has been defined as an independent risk factor for the subsequent development of BPD [103]. The generation of FR is one common pathway shared by these insults. Moreover, inadequate nutrition and how the baby is ventilated participate to the increase of OS [104].

“Old” BPD is characterized by a tissue remodeling process that has been divided into different phases, ending up in the chronic phase with an increased number of fibroblasts and fibrotic areas. Matrix metalloproteinases (MMPs) are important in fibrotic processes, and the balance between MMPs and their inhibitors normally drives the development of fibrosis. MMP expression is regulated at the transcriptional level by cytokines, growth factors, and extracellular matrix components. OS increases both MMPs and their inhibitors, thus causing a disruption of the extracellular matrix [104].

The “new” BPD was observed to have less fibrotic component and a more delayed alveolar development than the older counterpart [105]. Many authors suggested that an ongoing inflammatory process could be the prior mechanism in this “atypical” BPD [101, 106]. The release of inflammatory mediators can stimulate the endothelium to produce adhesion molecules, resulting in transendothelial cytokine migration [107]. The increased cytokine concentration could therefore enter in a “final common pathway” leading to OS-related lung damage, whether triggered by infection (antenatal or postnatal) or by lung stretching (airways, alveoli, basement membrane, and pulmonary capillary endothelium) [106]. Supporting this theory, there was an increased concentration of TNF-*α* and other proinflammatory cytokines in tracheal secretions of mechanical ventilated newborns with BPD. Phagocyte number and interleukin concentrations were also found to be elevated in bronchoalveolar lavage fluid of infants with BPD [108]. The phagocytic cells in the lung mediate their antimicrobial functions through the release of lysozymes, peroxidases, and proteases, but in addition, ROS and NO were released. Activated neutrophils and pulmonary type II cells are also important inducers of the Fenton reaction, which lead to a greater ROS generation [105].

### 3.4. Necrotizing Enterocolitis

Necrotizing enterocolitis (NEC), a syndrome of intestinal ischemic necrosis, is the most common gastrointestinal emergency in preterm infants and results in significant morbidity and mortality [109, 110]. NEC has a multifactorial etiology including low gestational age, low birth weight, low Apgar scores, hyaline membrane disease, formula feeding, umbilical vessel catheterization, and intestinal ischemia. Other risk factors are the prolonged antibiotic exposure, the sensitization to cow milk proteins, the genetic polymorphism in vascular endothelial grow factor, interleukin-10, and interleukin-12 [111–114]. Among them, a common synergistic effect of OS was described [115].

When epithelial gut cells are exposed to enteral feeding, the increased metabolic OS tips the population toward apoptosis, inflammation, bacterial activation, and eventual necrosis [116]. Mitochondria are the major source of intestinal apoptotic signaling, which is activated during OS condition [117]. OS also causes the partial inactivation of cyclooxygenase-1 (COX-1) and reduces the generation of gastroprotective prostaglandins (PG) that are known to inhibit gastric acid secretion, increase mucosal blood flow, and stimulate mucus-HCO_3_ secretion [118]. Ischemia and low-oxygen tension reduce the electron transport chain with subsequent excessive generation of hydroxyl radical (^•^OH), causing peroxidation of lipid cellular membranes and oxidative damage to proteins and other macromolecules. It is noteworthy that glutathione peroxidase (GPx), a major antioxidant enzyme in the gastric mucosa, was found to be inactivated during stress probably by excessively generated ^•^OH causing oxidative damage of GPx. This phenomenon seems to play a significant role in stress-induced gastric ulceration [119].

These triggering mechanisms result in significant inflammation of the intestinal tissues, release of inflammatory mediators, and downregulation of cellular growth factors. The proinflammatory cytokines activate a cascade of events leading to the eventual breakdown of the intestinal mucosal barrier and to severe NEC in some cases [120]. The immaturity of the gastrointestinal tract in preterm infants may also contribute to NEC development [121]. Toll-like receptor 4 (TLR4) expression is downregulated in the mature intestinal epithelium upon stimulation by Gram-negative lipopolysaccharide but is increased in the immature intestinal epithelium, leading to an exaggerated proinflammatory response through upregulation of the NF-*κ*B pathway [122]. Lipopolysaccharide from Gram-negative bacteria interacts with TLR4 expressed predominantly by enterocytes. This interaction results in the breakdown of the gut barrier and allows for pathogenic bacterial translocation. A proinflammatory response follows resulting in increased production of proinflammatory cytokines (IL-6, IL-8, and TNF) as well as increased Th17 cells and a decrease in the number of T regulatory cells. The combination of these cellular responses with TLR4 signaling results in a profound inflammatory response [123, 124]. Moreover, TLR4 decreases the expression of endothelial nitric oxide synthase (eNOS), thereby reducing the formation of nitric oxide (NO), an important vasodilator. All this results in intestinal ischemia and subsequent NEC.

We found a strong association between NEC and cord blood concentrations of AOPP, NPBI, and TH, showing a clear correlation between intrauterine OS events and the risk of developing NEC [125]. Ozdemir et al. reported a significant increase of intestinal malondialdehyde (MDA) in preterm infants with NEC [126]. All-trans retinoic acid treatment reduced the intestinal MDA elevation, suggesting an active lipid peroxidation in NEC disease [126]. Consistent with these results, the administration of antioxidant drugs has been shown to reduce intestinal mucosa damaged by ischemia or inflammation [127, 128]. TNF-*α* and IL-1*β* were reduced in animal model affected by NEC and treated with melatonin [129], a highly effective antioxidant and FR scavenger. Melatonin also showed beneficial effects as an adjuvant therapy in preterm newborns [130].

All these data establish the importance of perinatal oxidative insults in injured intestinal epithelial cells, thus proving a reasonable basis for developing new interventions to interrupt those mechanisms.

### 3.5. Renal Damage

The kidney is often severely damaged after asphyxia, but few data are available on renal oxidative damage in the newborn infant. Experimental studies show a high basal rate of aerobic metabolism in renal tissue, which suggests that the kidneys would be a primary target for this form of injury [131, 132]. The nephron of the mammalian kidney is composed of several cert types, each with distinct morphological, biochemical, and functional characteristics. One consequence of this heterogeneity is a different susceptibility to various forms of chemical and pathological injury. For example, medullary thick ascending limb cells and the pars recta of the proximal tubule are especially sensitive to injury after hypoxia or ischemia. FR-mediated lipid peroxidation has been implicated as a mechanism of tissue injury during ischemia. Lipid peroxidation products affect renal function directly by causing renal vasoconstriction or decreasing the glomerular capillary ultrafiltration coefficient and thus the glomerular filtration rate [133].

The activation of the phosphoinositide-3-kinase- (PI3K-) Akt-nuclear factor- (NF-) *κ*B axis was detected in this rat kidney ischemia reperfusion injury model [134]. A study by Weinberg et al. on hypoxic injury to isolated renal proximal tubules showed that exogenous GSH has a protective effect [135]. Renal GSH content is decreased by hypoxia or ischemia, and the decline is most rapid immediately after the cessation of blood flow [136]. Research on the glutathione system has shown that while total GSH levels tend to decrease, GSSG levels remain constant during the progression of chronic renal failure [137], suggesting that the kidney plays an important role in the maintenance of GSH concentrations in blood. Cellular responses to OS include the induction of heme oxygenase (HO) that helps to attenuate the adverse effects of these environmental factors [138].

The induction of HO and increased ferritin synthesis may be protective in renal oxidative injury, in which increased amounts of noncatalytic-free iron have a critical role or in circumstances in which oxidative cell injury is associated with increased intracellular content of heme. The release of iron from heme proteins contributes to oxidative kidney damage through hydroxyl radical generation and lipid peroxidation [139].

Increased HO activity allows the degradation of any heme released from endogenous oxidatively denatured heme proteins, whereas increased tissue ferritin-bound iron released from heme [140]. Administration of the iron chelator desferrioxamine during reperfusion is reported to limit postischemic renal dysfunction by an effect that appears to take place in the urinary space or along the adjacent brush border membrane. Markers of renal oxidative stress have been proposed [141]. Advanced oxidative protein products seem to be a reliable marker of the degree of oxidant-mediated protein damage and the potential efficiency of antioxidant therapy. Similarly, advanced glycation end products are enhanced during renal failure and are the result of the nonenzymatic reaction linking a protein amino group with a glucose-derived aldehyde group [142].

### 3.6. Oxidative Hemolysis

**Figure 3:**
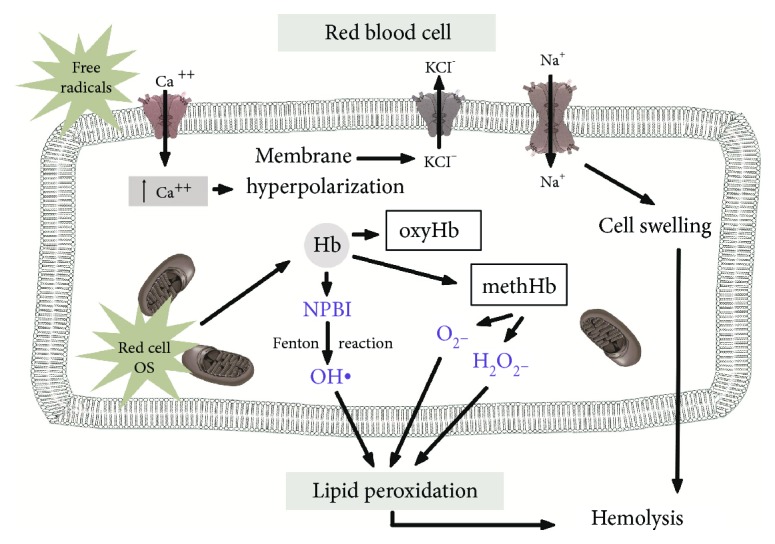
Schematic representation of oxidative red blood cell injury.

Red blood cells (RBC) have a wide array of antioxidant enzymes defending against attacks by FRs. Superoxide dismutase (SOD), catalase (CT), and glutathione peroxidase (GPX) represent great antioxidant resources of these cells against stressors associated with prematurity [143]. Otherwise, after exposure of RBCs to OS, a rapid loss of activity of age-dependent enzymes from reticulocytes occurs probably due to proteolysis. Various experiments of hypoxia or hypoxia-reoxygenation in vitro reported increased Heinz body formation, increased oxidation products of hemoglobin, and increased intraerythrocyte hydrogen peroxide generation suggesting the increased susceptibility of red blood cells to the oxidative damage (Figure 3) [144–146]. Our extensive investigations demonstrated the key role of OS and iron release in a reactive form via the Fenton reaction in the erythrocytes. When erythrocytes were incubated in medium containing oxidizing agents, iron release and the Fenton reaction led to the formation of the hydroxyl radical [147, 148]. Iron is released from hemoglobin or its derivatives, and the release is accompanied by methemoglobin formation. The iron diffused from erythrocytes into the incubation medium. Such diffusion, together with the higher susceptibility to release iron in newborn erythrocytes, could explain the appearance of plasma-free iron in newborns. Significant positive correlations were found between plasma-free iron and isoprostane levels in newborns, supporting the prooxidant role of free iron [149]. The higher free iron concentration, the higher lipid and protein peroxidation rate [150]. The erythrocytes are therefore a target of extracellular FR and at the same time generators of OS. The eryptosis should be considered as a cause of hemolysis when there is no evidence of an immune-mediated hemolytic anemia, no morphologic or laboratory data to suggest a problem of the red cell membrane, and no evidence of a quantitative or qualitative defect in hemoglobin synthesis in the newborns [146].

## 4. Conclusions

The existence of a redox homeostasis is essential for normal health and survival of the cell. When an unbalance between prooxidant and antioxidant factors occurs, OS is produced leading to cellular and tissue damage. The newborn, especially if preterm, is highly prone to OS and to the toxic effect of FRs. Taking advantage of the wealth of findings in perinatal OS researches, the relationship between OS-related mechanisms of injury and the so-called “free radical-related diseases of prematurity” can be considered irrefutable. Antioxidants may represent a very important weapon to fight this common way of damage. Collaterally, the development of biomarker OS panel has a very attractive prospect for future clinical application, in terms of prevention and response to therapy. Although much evidence already exists, more study is needed to eradicate the barrier hindering the widespread use of biomarkers. To conclude, it is no longer a long way from bench to bedside for antioxidant therapy and OS biomarkers in neonatology.

## Figures and Tables

**Table 1:** Antioxidants and diseases: clinical trials.

Disease	Antioxidant	Outcome	References
BPD	Lutein	Possible positive association between lutein and respiratory health	Melo van Lent, Leermakers; 2016
	iNO + vitamin A	Reduced the incidence of BPD and BPD + death and improved neurocognitive outcomes at 1 year in the 500–749 g birth weight group	Gadhia, Cutter; 2014
	Recombinant human SOD	Reduced early pulmonary injury, resulting in improved clinical status at 1 year corrected age Multiple intratracheal doses of rhSOD increase the concentration and activity of the enzyme in serum, tracheal aspirate, and urine	Davis, Parad; 2003 Davis, Rosenfeld; 1997
	Melatonin	Newborns who developed BPD had levels of IL-6, IL-8, TNF-alpha, and nitrite/nitrate values much higher than those in children who did not develop BPD	Gitto et al.; Pineal Res; 2004
	Vitamin A	Vitamin A supplementation does not reduce the incidence of BPD	Gawronski CA, Gawronski KM, Ann Pharmacother; 2016

NEC	L-Arginine	Enteral L-arginine supplementation appears to reduce the incidence of stage III NEC in VLBW infant	Polycarpou et al., 2013
	Pentoxifylline	Pentoxifylline did not change the risk of development of NEC in neonates with sepsis	Pammi et al., 2015
	Melatonin	Melatonin administration as an adjuvant therapy in neonatal NEC treatment is associated with improvement of clinical and laboratory outcome	[130]

ROP	Vitamin A	A trend towards reduced incidence of retinopathy of prematurity in vitamin A-supplemented infants	Darlow et al., Cochrane Database Syst Rev; 2002
	Vitamin E	There was a 52% reduction in the incidence of stage 3+ ROP in VLBW infants In VLBW infants, vitamin E supplementation significantly increased the risk of sepsis and reduced the risk of severe retinopathy and blindness	[94]; Brion et al., Cochrane Database Syst Rev; 2003
	D-Penicillamine	Six of the 70 surviving control infants and none of the 71 surviving treated infants had ROP stage II or greater Prophylactic enterally administered DPA does not prevent any stage ROP	Lakatos et al., Acta Paediatr; 1986 Tandon et al., Acta Paediatr; 2010
	Recombinant human SOD	rhSOD reduces the risk of developing ROP in extremely low gestational age newborn	Parad et al., Neonatology; 2012
	Lutein	There were no differences in the incidence of ROP at any stage between groups No significant effect was seen on threshold ROP	Romagnoli et al., J Matern Fetal Neonatal Med.; 2011 Manzoni et al., Am J Perinatol; 2013
	Allopurinol	Failure of allopurinol prophylaxis to prevent ROP	Russel et al., Arch Dis Child Fetal Neonatal Ed; 1995
